# Annotations

**Published:** 1895-11-16

**Authors:** 


### Annotations.

### A Mortal Calling.

The rate of mortality among medical men is becoming seriously high. Dr. Kortright has made an analysis of the ages and causes of death of 450 medical men who had spent their professional lives in New York and its vicinity. Those men, practising the healing and life-saving art upon others, only themselves reached an average of 54·6 years. Whereas among lawyers the mortality figures stood at 20·23, and among clergymen were so low as 15·93, it stood at 25·53 among the doctors. In short, according to Dr. Kortright's figures, doctors are the least long-lived of all classes except publicans, butchers, factory operatives, and quarrymen. An appalling fact to be added to these is that suicide is four times as common among doctors as among other full-grown men. The reasons for this state of things are well known to medical men, and chief among them all is the worrying and anxious nature of the profession. The worry and anxiety of the doctor, combined with his too often miserable remuneration, produce sleeplessness, brain weariness, mental incapacity, and too often a depression and anguish of mind which can hardly be paralleled in any other calling. And yet, in this country, because the doctor is respected, innumerable young men rush in every year, many of whom are from the first foredoomed to failure, melancholy, and suicide or other premature death. In the interests of the thousands who are entering the profession every year, responsible leaders should make known far and wide facts so full of significance to every member of the middle-class who can influence the choice of a profession by the inexperienced and the young.

### The Dangers of Adulterated Boots.

Leather, a morning contemporary tells us, is becoming very dear, and, by consequence, inferior boots are now being put upon the market. We are told of "adulterated boots," in which "glucose" and "curriers' shavings" play a conspicuous part. Certain classes of cheap boots are said to be made up with "a species of spongy diaphragm of curriers' shavings between the inner and the outer sole," and the "spongy diaphragm if once damped will never dry." That means at least two things: namely, footgear which prevents the free transudation of perspiration, and which, therefore, causes the feet to smell; and, what is far more important, it ensures the constant application of a cold damp surface to the sole of the foot. Now, cold and damp feet will produce in some persons catarrh, in some sore throats, in many colic, and in a few peritonitis and death. All these things demand consideration at the hands of sensible people. Obviously the boots most likely to have a "spongy diaphragm, which once wetted will never dry," are cheap ones, and most probably are "ready made." What we ought all to do is to search out thoroughly honest shoemakers and have our boots made to order. It may help to keep the said shoemakers up to the high-water mark of honesty if we show them that we understand something about the matter, and are able to detect for ourselves any attempt at "adulteration" on their part. In any case, everybody should avoid boots which "once wet never dry again" exactly as they would avoid bad drains or any other known danger to health and life.

### Our Homeless Ones.

The difficulty of the casual is, in country places, mostly bound up with the difficulty of the tramp. But it is otherwise in towns. Mod ern methods of pro duction tie each man down more and more to one special line of work, and the constant oscillations of trade tend more and more frequently to deprive him for uncertain periods of the particular sort of work which he is alone able to perform. Whether we will or no, therefore, it must be the case that, as a part of the very civilisation of which we are so proud, there must constantly be a multitude of people in dire distress. This is not an affair of one country or of one season, nor is the provision of "shelters" a mere phase of fanciful philanthropy. It is a necessary outcome of modern methods of division of labour. Our reason for insisting upon this is that if the provision of night shelters is a necessity, equally necessary also is their control by legal authority. Both the common lodging-houses and the casual wards are under supervision, but sandwiched in between these are the so-called philanthropic "shelters," over which the police have no control and into which the sanitary officials can only penetrate by special order given after application before a magistrate. In Berlin the same difficulty is felt, and there a shelter has been established by the municipality. The greater portion of the shelter is intended for the reception of single men and women who merely pass the night there. All on entering must have a bath, and during this operation their clothes are disinfected by steam. After the bath they receive soup and bread. The dormitories are warmed by steam, which reduces to a minimum the amount of bed-clothes required, and all the coverlets are disinfected by steam before being used again. Accommodation is thus provided for nearly three thousand people every night, the cost per head is about one penny, and by the compulsory cleansing of clothes and person the shelter, instead of a danger becomes a distinct sanitary advantage to the community. Compare this with our so-called charitable shelters! We repeat, the provision of shelter for a large number of homeless ones is a necessity of the present phase of our civilisation. Many of these people can pay something for their lodging, many of them must of necessity be dealt with by charity or the poor law; but, whether they pay or not, wherever masses of people are so brought together in haphazard fashion, means should be taken to prevent such aggregations becoming a sanitary danger. "Shelters" should at the least provide as good accommodation as the common lodging-house on the one hand, or the casual ward on the other, and, what is equally important, should be quite as much open to inspection as either of these places.

